# In silico analysis of the immune microenvironment in bladder cancer

**DOI:** 10.1186/s12885-020-06740-5

**Published:** 2020-03-30

**Authors:** Ye Zhang, De-hua Ou, Dong-wu Zhuang, Ze-feng Zheng, Ming-en Lin

**Affiliations:** 1 Department of Urology, The First Affiliated Hospital of Shantou University Medical College, No. 57, Changping Road, Jinping District, Shantou, Guangdong China; 2 Shantou University Medical College, No. 22, Xinling Road, Jinping District, Shantou, Guangdong China

**Keywords:** Immune cell infiltration, Microenvironment, Bladder cancer, Survival

## Abstract

**Background:**

Infiltrating immune and stromal cells are vital components of the bladder cancer (BC) microenvironment, which can significantly affect BC progression and outcome. However, the contribution of each subset of tumour-infiltrating immune cells is unclear. The objective of this study was to perform cell phenotyping and transcriptional profiling of the tumour immune microenvironment and analyse the association of distinct cell subsets and genes with BC prognosis.

**Methods:**

Clinical data of 412 patients with BC and 433 transcription files for normal and cancer tissues were downloaded from The Cancer Genome Atlas. The CIBERSORT algorithm was used to determine the relative abundance of 22 immune cell types in each sample and the ESTIMATE algorithm was used to identify differentially expressed genes within the tumour microenvironment of BC, which were subjected to functional enrichment and protein-protein interaction (PPI) analyses. The association of cell subsets and differentially expressed genes with patient survival and clinical parameters was examined by Cox regression analysis and the Kaplan-Meier method.

**Results:**

Resting natural killer cells and activated memory CD4^+^ and CD8^+^ T cells were associated with favourable patient outcome, whereas resting memory CD4^+^ T cells were associated with poor outcome. Differential expression analysis revealed 1334 genes influencing both immune and stromal cell scores; of them, 97 were predictive of overall survival in patients with BC. Among the top 10 statistically significant hub genes in the PPI network, *CXCL12*, *FN1*, *LCK*, and *CXCR4* were found to be associated with BC prognosis.

**Conclusion:**

Tumour-infiltrating immune cells and cancer microenvironment-related genes can affect the outcomes of patients and are likely to be important determinants of both prognosis and response to immunotherapy in BC.

## Background

Bladder cancer (BC) is a complex disease characterized by high morbidity and mortality; thus, 81,190 newly diagnosed cases and 17,240 deaths were reported in the USA in 2018 [1]. Among the patients with BC, approximately 25% have muscle-invasive cancer or metastatic disease and 75% have non-muscle invasive cancer (NMIBC) [2]. Although the proportion of NMIBC is relatively high, the key clinical concerns for these patients are a high recurrence rate (70%) in those with low- and intermediate-risk disease and a relatively high rate of progression to muscle-invasive cancer (30%) in those with high-risk disease [3–5].

The tumour microenvironment (TME) surrounding cancer cells originally consists of tumour stromal cells, the extracellular matrix, and soluble molecules. Once the TME is formed, many immune cells such as T cells, medullary inhibitory cells, and macrophages, infiltrate the TME through chemotaxis, further contributing to its composition. Thus, the two main non-tumour components of the TME are immune cells and stromal cells. Increasing evidence indicates that the tumour phenotype is shaped not only by the intrinsic properties of cancer cells, but also by the activity of immune cells in the TME [6]. Furthermore, immune infiltration into the tumour site has been associated with both overall survival (OS) and treatment response in such types of cancer as colorectal cancer, breast cancer, and liver cancer [7–9].

Despite significant advances in understanding cancer biology, including the functional role of the TME, the treatment of patients with BC still remains challenging. As migration of immune cells into the tumour site is closely related to clinical results and disease outcome, these cells could be used as drug targets to improve survival of patients with BC [10–12]. However, immunophenotyping in cancer could be problematic as the existing experimental methods such as immunochemistry require multiple biomarkers and can miss certain cell populations. In this respect, high-throughput approaches to cell typing and gene expression profiling may offer a solution because they enable analysis of multiple data independent of collection time or site, or performance of biomarkers.

CIBERSORT is a versatile computational method for quantifying cell fractions from bulk tissue gene expression datasets based on immune cell signatures. By combining an approach called support vector regression with the knowledge of expression profiles of 22 human haematopoietic cell subsets comprising ~ 500 marker genes, CIBERSORT could quantify the relative proportion of each cell type [13, 14]. The ESTIMATE (Estimation of STromal and Immune cells in MAlignant Tumour tissues using Expression data) method integrates publicly available datasets such as The Cancer Genome Atlas (TCGA) and can be applied to predict general fractions of immune and stromal cells in a tumour as well as tumour purity in a sample based on cell genetic signatures [15–17].

In this study, we used the ESTIMATE and CIBERSORT analytical methods to determine individual immune cell profiles in the TME of BC samples according to specific characteristics of each cell subset. The knowledge regarding the infiltration of immune cells into tumours could be used in personalized medicine to reveal individual drug targets, which should improve the survival of patients with BC.

## Methods

### Data mining using TCGA cohort

The data from TCGA (https://tcga-data.nci.nih.gov/tcga/) downloaded in April 2019 included a total of 433 transcription files (19 normal tissues and 414 BC samples) and clinical characteristics of 412 patients with BC. Only patients diagnosed with BC, for whom clinicopathological data and survival information were available, were included. The following demographic and clinical data were extracted: sex, age, survival status, topography, and lymph node and metastasis (TNM) stage based on the American Joint Committee on Cancer (AJCC). Patients with missing or insufficient data were excluded from subsequent analysis.

The TME was assessed in 414 BC samples using the ESTIMATE package in R (version 3.5.2, https://www.r-project.org). Gene expression datasets were prepared using standard annotation files and uploaded to the CIBERSORT web portal (http://cibersort.stanford.edu/), with the algorithm based on the default signature matrix at 1000 permutations. After converting the gene expression matrix into the immune cell matrix (433 transcription files) and applying the filtering criteria for gene transcription (*P* < 0.05) in CIBERSORT (Perm = 1000), 162 samples (5 normal tissues and 157 tumours) were selected to visualize the matrix of 22 immune cell fractions.

### Visual display of 22 immune cell types

The matrices of 22 immune cell subsets, their correlations, and gene expression profiles were presented as barplots, heat maps, and violin maps using R packages pheatmap, corrplot, and vioplot (https://www.r-project.org).

### Evaluation of BC-infiltrating immune cells and the TME

ESTIMATE is a tool for predicting tumour purity and the presence of infiltrating stromal/immune cells in the TME based on gene expression data. The ESTIMATE algorithm is based on single-sample Gene Set Enrichment Analysis (ssGSEA) and generates three scores: stromal cell scores, immune cell scores, and ESTIMATE scores (which have higher correlation with tumour purity compared with stromal-only and immune-only scores). CIBERSORT is a deconvolution algorithm that can estimate the cellular composition of complex tissues based on standardized gene expression data and quantify the abundance of specific cell types. CIBERSORT derives a *P* value for the deconvolution of each sample using Monte Carlo sampling, thus providing a measure of confidence in the results of the inferred immune cell fractions; therefore, only samples with a CIBERSORT *P* < 0.05 were considered eligible for further analysis. The proportions of immune cells were predicted separately for each gene expression series; the sum of different immune cell fractions in each sample equalled 1.

### Identification of differentially expressed genes (DEGs)

The samples were divided according to the scores of stromal and immune cells: those with scores below the median value were assigned to a low-score group, whereas those with scores equal or above the median were assigned to a high-score group. Data analysis was performed using the R limma package. Fold change (FC) > 1 and false discovery rate (FDR) < 0.05 were set as the cut-off criteria to screen for DEGs. The heatmap of the DEGs was drawn using the R pheatmap package; DEGs with the same signatures were clustered together, indicating their specificity.

### Gene ontology (GO) and Kyoto encyclopedia of genes and genomes (KEGG) enrichment analysis

GO analysis was applied to explore functions of the identified DEGs by organizing genes into hierarchical categories of biological process, molecular function, and cellular component. KEGG pathway analysis was performed to reveal the functions and interactions among the DEGs based on the enrichment ratio of the sequenced gene to all annotated genes in the pathway. Data analysis was performed using stringi and ggplot2 packages in R (https://www.r-project.org). *P* < 0.05 was set as the cut-off criterion indicating significant enrichment of functional GO terms and KEGG pathways.

### Identification of protein-protein interactions (PPIs) of DEGs

Identification of protein complexes and functional modules was performed by constructing PPI networks using an online database resource Search Tool for the Retrieval of Interacting Genes (STRING; https:// string-db.org), which provides comprehensive coverage of experimental and predicted protein interactions with the confidence of custom value > 0.96. The obtained PPI networks were visualized using Cytoscape version 3.6 (https://cytoscape.org).

### Association of patient OS with immune cell fractions and DEGs

Cases with a CIBERSORT *P*-value of < 0.05 were included in survival analysis. Median values of the proportions of each cell subset were computed and used to determine the correlation between immune cell types and patient outcome by Cox regression analysis. Kaplan-Meier curves were generated to reveal the correlation between patients’ OS and DEG levels, which was examined by log-rank test.

### Expression of immunomodulatory factors

Expression levels of several key immunomodulatory factors such as lymphocyte-activation gene 3 (LAG-3), hepatitis A virus cellular receptor 2 (HAVCR2), cytotoxic T-lymphocyte-associated protein 4 (CTLA-4), interferon-γ (IFN-γ), inducible T-cell costimulator (ICOS), Intercellular Adhesion Molecule 1 (ICAM-1), T cell immunoreceptor with Ig and ITIM domains (TIGIT), programmed cell death protein 1 (PDCD1/PD-1), programmed death-ligand 1 (PDL-1/CD274), NKG2-C type II integral membrane protein (KLRC1), and V-set immunoregulatory receptor (VSIR) were quantified in normal bladder tissues and BC tissues. Differences in gene expression between normal and BC tissues and between high-score and low-score groups were analysed by *t*-test.

## Results

### Performance of ESTIMATE and CIBERSORT

**Fig. 1:**
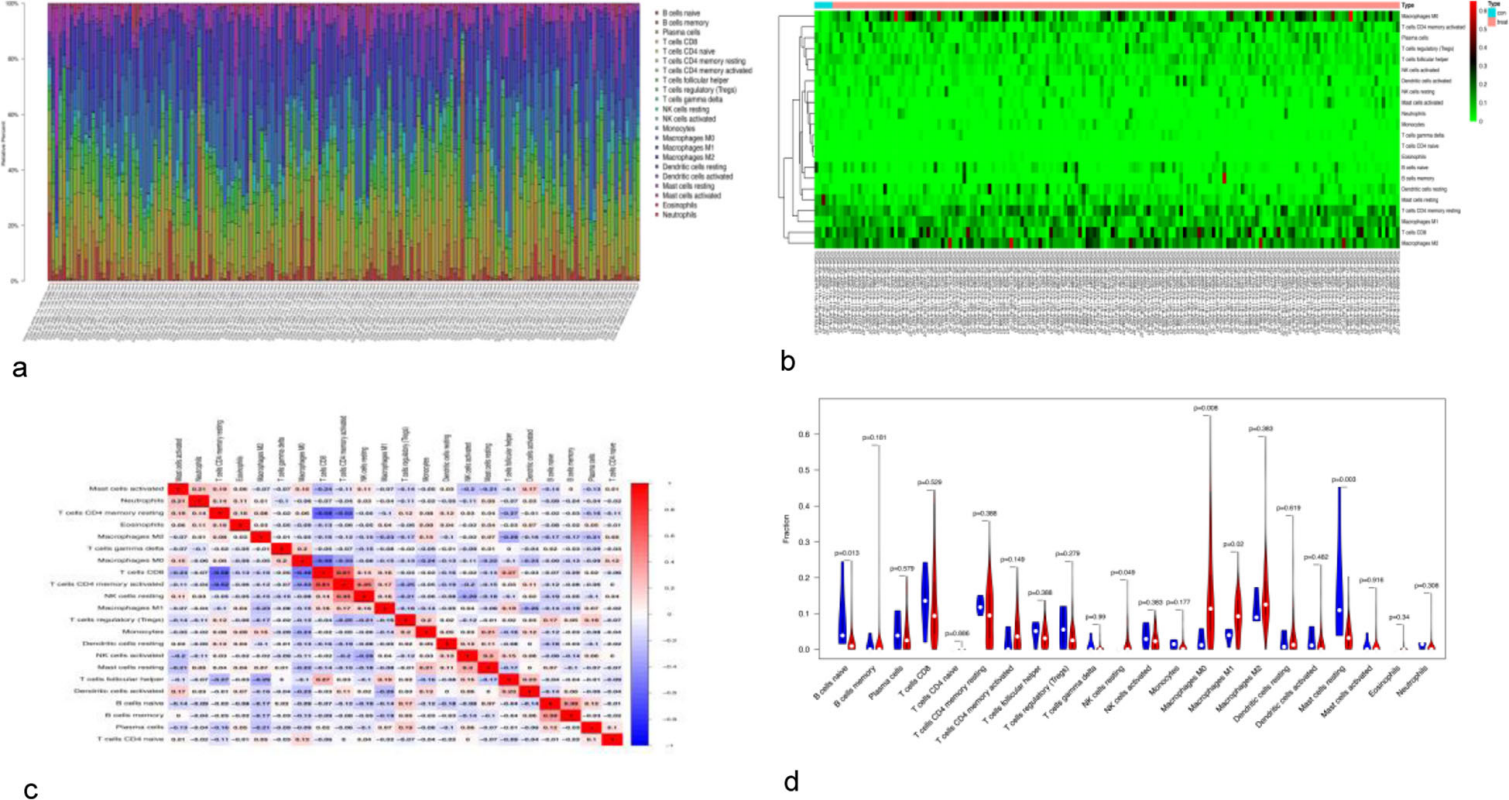
Immune cell subsets in BC analysed using CIBERSOST. **a** A bar chart displaying proportions of immune cell subsets. The X-axis shows sample names and the Y-axis shows percentages of 22 immune cell types, which were predicted separately for each gene expression series. **b** A heat map of the proportions of 22 immune cell types. Sample names and classification are shown below, sample clustering is shown on the left, and 22 immune cell types are indicated on the right. **c** Correlation matrix of 22 immune cell types. Variables were organized by average linkage clustering. Red and blue colours indicate positive and negative correlation, respectively; colour intensity corresponds to the degree of correlation. **d** A violin map of 22 immune cell types. The X-axis shows cell types and the Y-axis indicates fractions; blue and red colours represent normal and cancer tissues, respectively

We downloaded 433 transcription files, including 19 for normal tissues and 414 for BC tissues, and clinical information of 412 patients from TCGA database. The 414 tumour files were graded by ESTIMATE, and stromal cell scores, immune cell scores, and ESTIMATE scores were computed. The gene expression matrix (433 files) was converted into the immune cell matrix and combined with the composition and percentages of immune cells using CIBERSORT. Based on the screening cut-off criterion of *P* < 0.05, we obtained 162 (5 normal and 157 tumour) statistically significant immune cell matrices and visualized them using barplot, heat maps, correlation heat maps, and violin diagrams. Analysis of cellular characteristics showed that tumour-related macrophages were the most abundant TME-infiltrating cells, followed by CD4-positive T cells, and plasma cells. Macrophages of MO, M1, and M2 states showed low presence in normal tissues and high presence in cancer tissues (Fig. 1a, b). The correlation heat map revealed that CD8^+^ T cells and activated memory CD4^+^ T cells were negatively correlated with resting memory CD4^+^ T cells, whereas activated memory CD4^+^ T cells were positively correlated with CD8^+^ T cells and resting natural killer (NK) cells (Fig. 1c). The violin map showed that there were more intuitively resting memory CD4^+^ T cells, CD8^+^ T cells, and macrophages in cancer than in normal tissues (Fig. 1d), accounting for their increased proportions. M0 and M1 macrophages and resting NK cells showed high abundance in tumours but low abundance in normal tissues; in contrast, naive B cells and resting mast cells showed high abundance in normal tissues and low abundance in cancerous tissues.

### Gene expression profiling in BC samples depending on immune and stromal cell scores

**Fig. 2:**
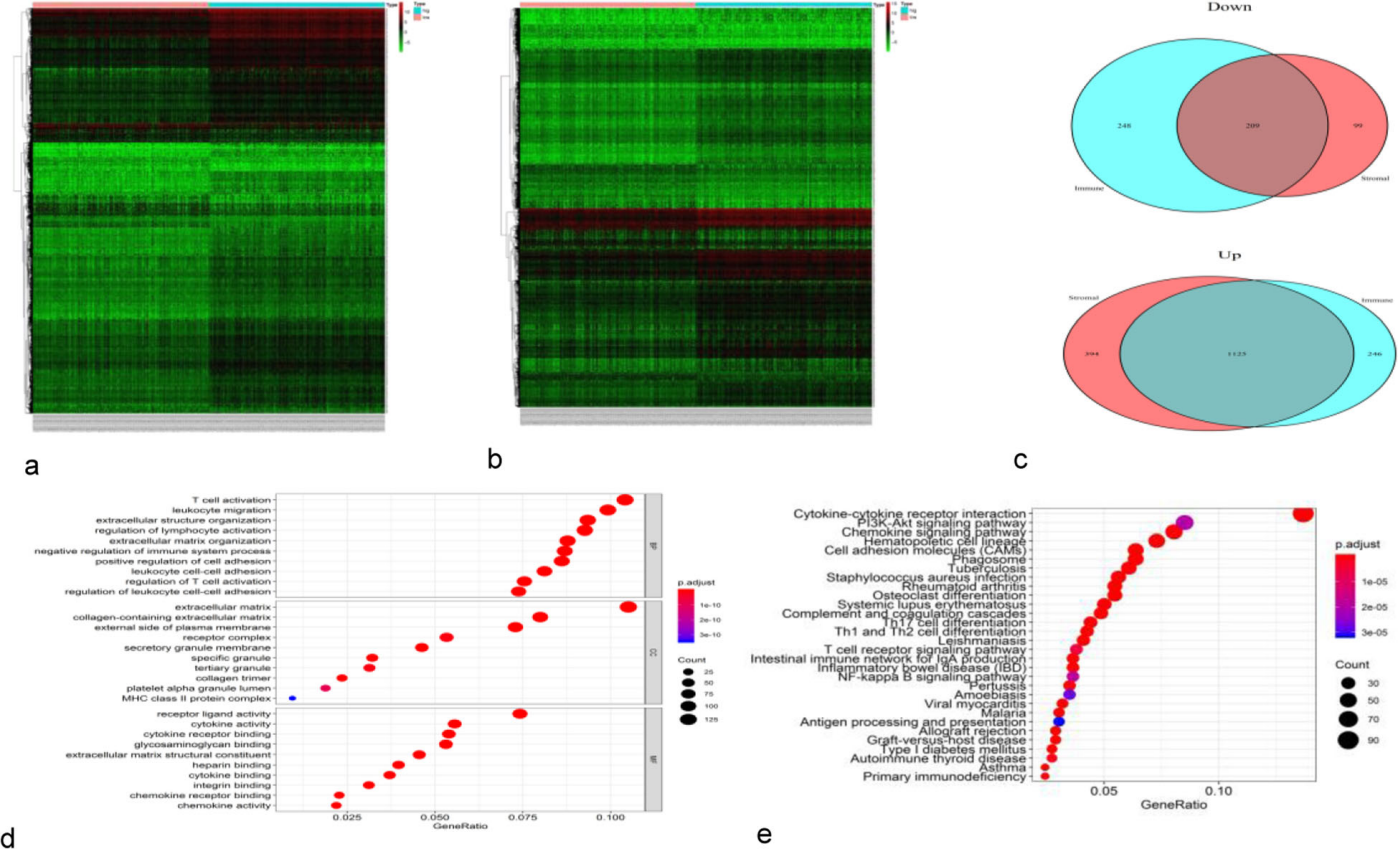
Analysis of DEGs in samples with high and low stromal and immune cell scores. Patient samples were divided into low- and high-score groups. Heatmaps of the DEGs depending on the stromal (**a**) and immune (**b**) cell scores. DEGs commonly downregulated and upregulated in the high-score groups (**c**). Top 10 GO terms (**d**) and top 30 KEGG terms (**e**) of the 1334 commonly regulated DEGs. The spot size indicates the number of DEGs enriched and the spot colour indicates the level of significance

To reveal the correlation of gene expression profiles with immune and stromal cell scores, the samples were divided into low- and high-score groups. Comparison of the two stromal score groups revealed 1827 DEGs corresponding to the cut-off criteria (log FC > 1, *P* < 0.05); among them, 1519 and 308 were significantly upregulated and downregulated, respectively, in the high-score group. Comparison of the two immune score groups revealed 1371 upregulated and 457 downregulated DEGs. The heat map constructed using unsupervised hierarchical clustering analysis showed that the DEGs in the low- and high-score groups could be clearly separated (Fig. 2a, b). The Venn diagram revealed 1125 and 209 DEGs commonly upregulated and downregulated, respectively, in samples with high scores for immune and stromal cells (Fig. 2c).

### Functional characteristics of the identified DEGs

To predict the functions of the 1334 DEGs identified in high-score BC samples, we performed GO enrichment and KEGG pathway analyses. The top 10 GO categories associated with the DEGs were: T cell activation, leukocyte migration, and negative regulation of the immune system (biological processes), extracellular matrix (cellular components), and receptor-ligand activity, cytokine activity, and glycosaminoglycan binding (molecular functions) (Fig. 2d). Among the KEGG pathways, the DEGs were enriched in cytokine-cytokine receptor interaction, PI3K/AKT signalling pathway, and chemokine signalling pathway (Fig. 2e).

### PPI network of common DEGs

**Fig. 3:**
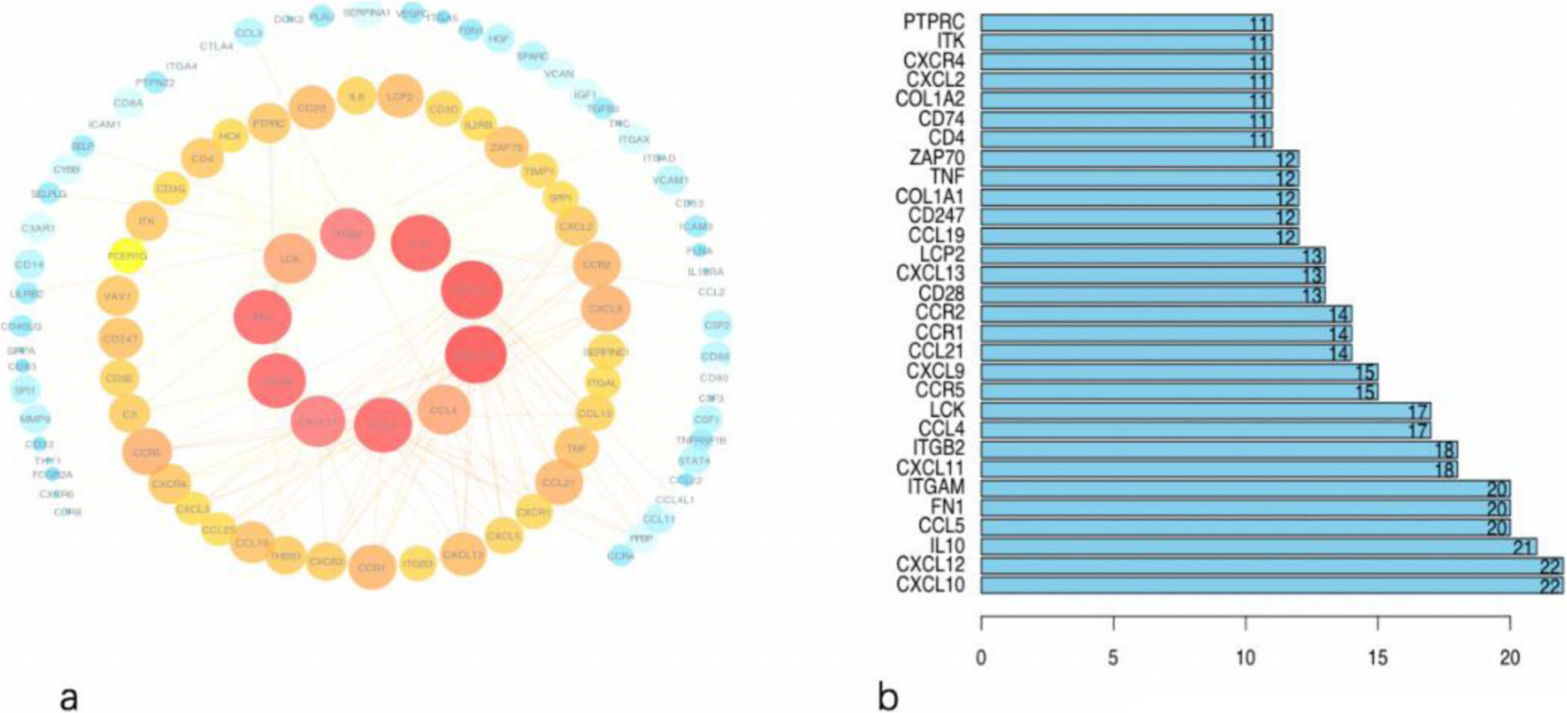
Analysis of the PPI network. **a** The PPI diagram; node colour reflects the log FC of gene expression and node size indicates the number of interacting proteins. **b** A histogram showing numbers of top-ranked connection nodes for the indicated genes

To better understand the interplay among the identified DEGs, we constructed the PPI network using STRING, which revealed that the DEGs were densely interconnected. The top 10 hub genes in the PPI network were *CXCL10*, *CXCL12*, *IL10*, *CCL5*, *FN1*, *ITGAM*, *CXCL11*, *ITGB2*, *CCL4*, and *LCK* (Fig. 3).

### Association of imm Une cell subsets and DEGs with BC outcomes

**Fig. 4:**
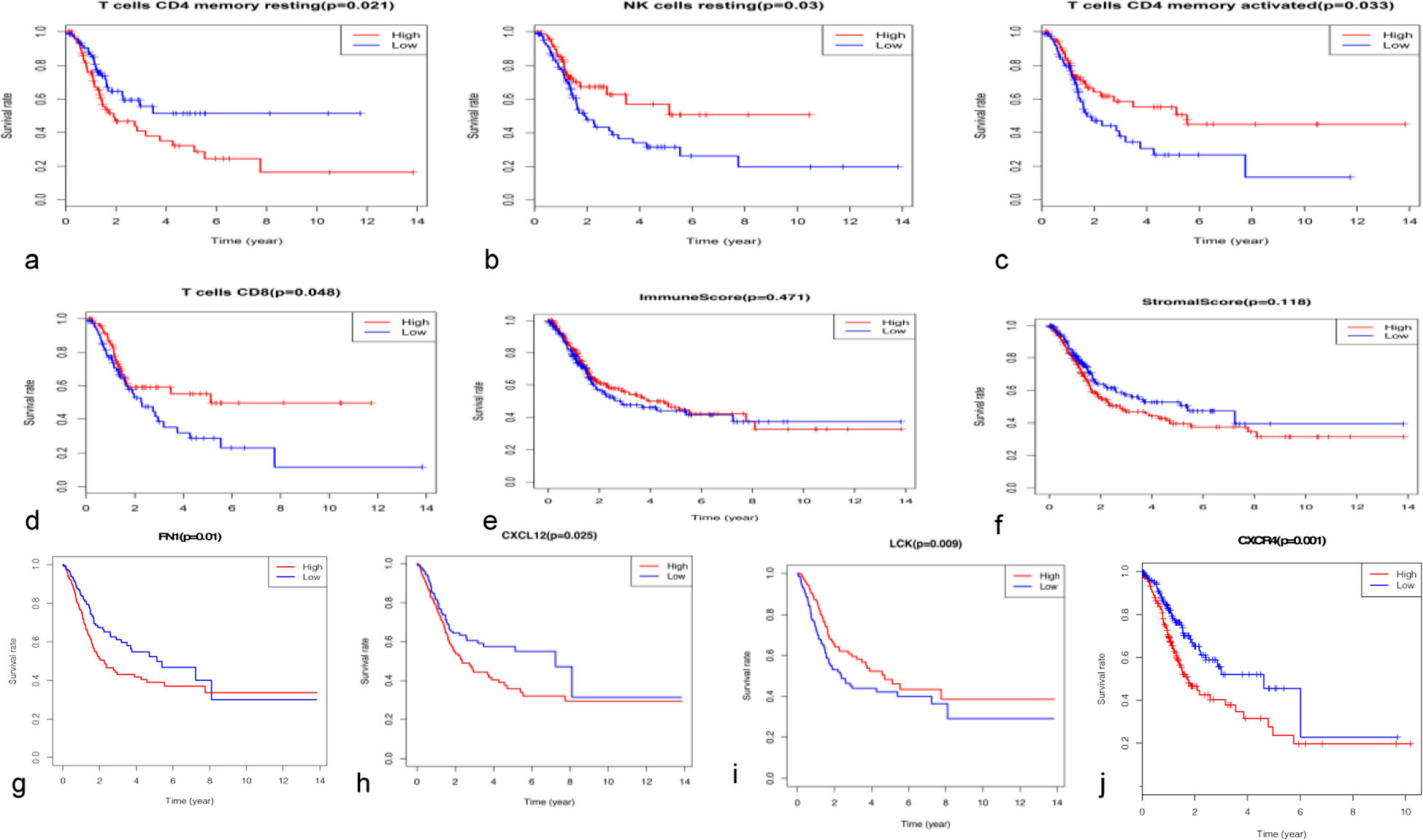
Association of immune cell subsets and DEGs with patient survival. Kaplan-Meier survival curves were generated by dividing patients into groups with high (red lines) and low (blue lines) abundance of immune cell types or expression of DEGs from the PPI network. Graphs show OS according to the presence of CD4^+^ resting memory T cells (**a**), resting NK cells (**b**), activated memory CD4^+^ T cells (**c**), and CD8^+^ T cells (**d**)**,** immune cell scores (**e**) and stromal cell scores (**f**), and the expression of FN1 (**g**), CXCL12 (**h**), LCK (**i**), and CXCR4 (**j**)

Next, we matched the immune cell matrix with the clinical survival time and cancer stage. The results indicated that OS of patients with BC was significantly negatively associated with resting memory CD4^+^ T cells and positively associated with resting NK cells, activated memory CD4^+^ T cells, and CD8^+^ T (Fig. 4a–d). There was no statistically significant association of OS with the immune cell score (*P* = 0.471) or stromal cell score (*P* = 0.118), although the latter showed a tendency to correlate with shorter OS (Fig. 4e, f).

Survival correlation analysis of the 1334 DEGs revealed that 97 genes were significantly associated with patient OS (*P* < 0.01). The top 10 DEGs were: *GPR25* (*P* = 7.97E-06), *CYP4F12* (*P* = 3.60E-05), *MAP 1A* (*P* = 4.22E-05), *HOXB3* (*P* = 7.11E-05), *SMAD6* (*P* = 0.00012), *EPHB6* (*P* = 0.00013), *CPA4* (*P* = 0.00014), *CASQ2* (*P* = 0.00015), *HSPB6* (*P* = 0.00016), and *LRRC32* (*P* = 0.0002).

Among the top 10 hub genes in the PPI network, the genes encoding fibronectin 1 (*FN1*), C-X-C motif ligand 12 (*CXCL12*), lymphocyte-specific protein tyrosine kinase (*LCK*), and C-X-C chemokine receptor type 4 (*CXCR4*) were significantly associated with patient OS (Fig. 4g–j).

**Fig. 5:**
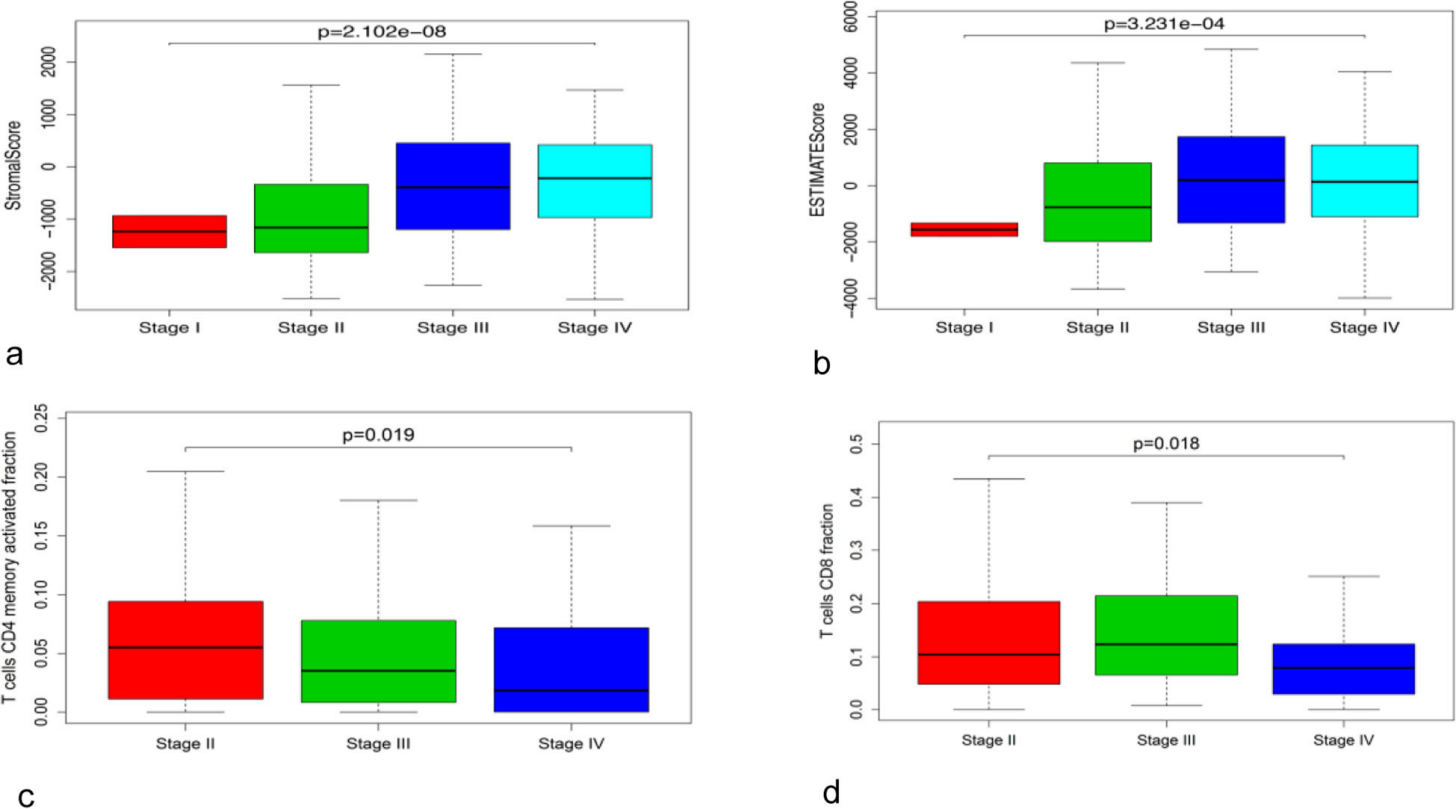
Correlation of BC clinical stages with stromal (**a**) and ESTIMATE (**b**) scores and with the abundance of activated memory CD4^+^ T cells (**c**) and CD8^+^ T cells (**d**)

**Fig. 6:**
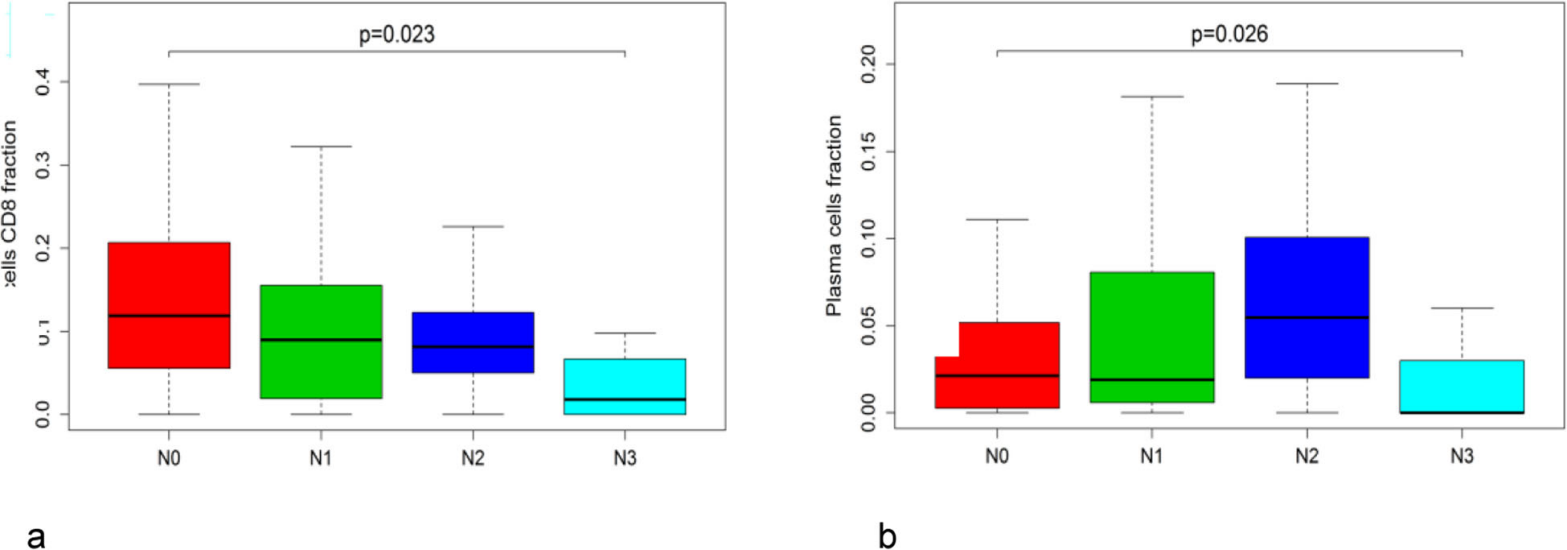
Association of the number of metastatic lymph nodes (N) with CD8^+^ T cells (**a**) and plasma cells (**b**)

The stromal cell score and the ESTIMATE score were positively correlated with the BC stage (Fig. 5a, b), indicating that the purity of tumour cells decreased with cancer progression. We also observed that the levels of activated memory CD4^+^ and CD8^+^ T cells decreased with the BC stage (Fig. 5c, d) and that CD8^+^ T cells and plasma cells showed a statistically significant reduction in the N3 stage (Fig. 6a, b).

Univariate Cox regression analysis revealed that resting memory CD4^+^ T cells were significantly associated with better outcome (hazard ratio [HR] = 0.562, 95% confidence interval [CI] = 0.343–0.922; *P* = 0.023), whereas CD8^+^ T cells (HR = 1.634, 95% CI = 0.999–2.672; *P* = 0.05), activated memory CD4^+^ T cells (HR = 1.704, 95% CI = 1.039–2.795; *P* = 0.035), and resting NK cells (HR = 1.749, 95% CI = 1.047–2.921; *P* = 0.033) showed association with poor outcome (Additional file 1).

### Expression profile of immunomodulatory genes

**Fig. 7:**
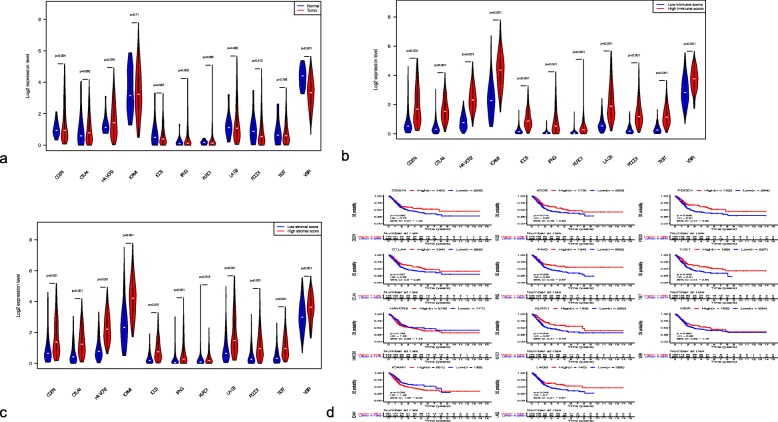
Expression levels of immunomodulatory genes and their association with patient survival. Differences in gene expression between normal and BC tissues (**a**) and between samples with low and high immune cell scores (**b**) and stromal cell scores (**c**). Kaplan-Meier curves showing correlation of OS with expression levels of 11 immunomodulatory genes (**d**)

*CD274*, *HAVCR2*, and *IFNG* were significantly upregulated in BC samples compared with normal tissues (Fig. 7a). The expression of 11 genes encoding immunomodulatory factors (*LAG3*, *HAVCR2*, *CTLA4*, *IFNG*, *ICOS*, *ICAM1*, *TIGIT*, *PDCD1*, *CD274*, *KLRC1*, and *VSIR*) was significantly increased in the groups with high stromal and immune cell scores (Fig. 7b, c). Analysis of the prognostic value of these genes indicated that patients with high expression of *LAG3*, *CTLA4*, *IFNG*, *ICOS*, *TIGIT*, *PDCD1*, and *KLRC1* and low expression of *ICAM1* had longer OS (Fig. 7d).

## Discussion

To improve the prognosis of BC, it is essential that patients should be regularly checked for cancer recurrence or progression, which may depend on the infiltration of immune cells into the tumour site. However, the immune mechanisms involved in the occurrence and progression of BC are not fully elucidated and it is unclear which immune cells or factors are the most prognostically significant.

In this study, we performed TCGA data mining to reveal the correlation between the infiltration pattern of immune cells into the TME and clinical characteristics of patients with BC. CIBERSORT was used to calculate the proportions of 22 immune cell subsets in the tumour transcriptome, and ESTIMATE was applied to evaluate the fractions of immune and stromal cells, which were then analysed for correlation with cancer advancement and patient survival. Our results showed that stromal cell scores were positively correlated with cancer stage, indicating that stromal components in the TME may play an important role in BC progression, which is consistent with the findings of a previous study [18]. Indeed, the tumour stromal components are known to contributes to cancer budding, epithelial-mesenchymal transformation, and lymph node metastasis [19, 20], which may account for their association with cancer progression.

Although we did not observe a direct correlation between the stromal/immune cell scores and patient survival, different subsets of immune cells showed significant association with the BC outcome. Thus, the increase in resting memory CD4^+^ T cells was significantly associated with better outcome, whereas that in CD8^+^ T cells, activated memory CD4^+^ T cells, and resting NK cells was correlated with poorer outcome. A previous study showed that a significant reduction in the number of CD4^+^ and CD8^+^ tumour-infiltrating lymphocytes (TILs) during non-classical differentiation in advanced BC may be associated with lower tumour immunogenicity and immune tolerance towards cancer and that a decrease in CD4^+^ TILs was indicative of poor prognosis [21]. Similarly, patients with advanced urothelial carcinoma (pT2, pT3, or pT4) who had higher numbers of CD8^+^ TILs (> 8), showed longer disease-free survival (*P* < 0.001) and OS (*P* < 0.018) compared to those who had fewer CD8^+^ TILs [22]. These findings indicate that in advanced BC, the levels of CD4^+^ and CD8^+^ cells decrease, negatively affecting disease prognosis, which is consistent with our observations that activated memory CD4^+^ cells, CD8^+^ T cells, and plasma cells decreased with the increase of cancer stage and lymph node metastasis. However, it was difficult to detect a trend for clinical improvement, because among the 412 samples analysed only two had BC stage 1 and there were no matching transcription files. Nevertheless, this fact did not affect our prognostic results, which support the notion that only certain subsets of tumour-infiltrating immune cells have a potential to predict clinical outcomes. Thus, the CD4^+^ cell population as a whole cannot be considered for BC prognosis, because its subsets showed the opposite trends: activated memory CD4^+^ T cells were associated with better outcome, whereas resting memory CD4^+^ T cells – with poorer outcome.

We also identified DEGs in samples with different stromal/immune cell scores and analyzed their potential functional activity, PPI, and association with patient prognosis. The 1334 common genes differentially expressed in both stromal and immune cell high-score groups were enriched in such GO categories as T cell activation, leukocyte migration, negative regulation of immune system, and the extracellular matrix. Pathway analysis revealed enrichment of DEGs in KEGG pathways of cytokine-cytokine receptor interaction, and PI3K/AKT and chemokine signalling. Consistent with these results, previous studies have demonstrated that immune system functions are critical for the formation of a complex BC microenvironment [23, 24], which may explain our finding that 97 DEGs were significantly associated with patient survival.

PPI analysis revealed that the top 10 hub genes in the BC microenvironment were related to cytokines, chemokines, and their receptors, which is in agreement with the role of cytokines and chemokines in shaping the TME [25, 26]. Four of the hub genes, *CXCL12*, *LCK*, *FN1*, and *CXCR4*, were found to be associated with patient survival. As CXCL12 is the second highest interconnected node in the PPI network negatively associated with OS, it deserves more attention. CXCL12, which belongs to the C-X-C family, binds to CXCR4 and triggers various immunological effects, including stimulation of monocyte, NK, and T cell migration and changes in protein expression. CXCL12 can potentially serve as a prognostic factor for gastrointestinal malignancies, including hepatocellular carcinoma and pancreatic cancer [27–29]. CXCR4, which is upregulated during BC progression, interacts with CXCL12 in cancer cells to mediate tumour chemotaxis and invasion through connective tissue, suggesting that CXCR4 may be a potential target for attenuation of BC metastasis [30]. Our results indicate that CXCR4 and its ligand CXCL12 may not only serve as prognostic indicators in BC but may also play a role in the PPI network involved in cancer progression. Chemokines and their receptors control cancer development through regulation of leukocyte infiltration, tumour-related angiogenesis, tumour-specific host immune responses, and cancer cell proliferation and migration [31]. Although the molecular mechanisms underlying cancer metastasis remain to be fully elucidated, accumulating evidence points on a significant role of CXCL12/CXCR4 in the process [32–35], suggesting that the CXCL12/CXCR4 axis may be a potential therapeutic target in BC.

FN1 is an extracellular matrix component involved in a variety of cellular processes, including carcinogenesis [36, 37]. Several studies have reported that FN1 modulates cell behaviour through interaction with integrin ITGA5 and activation of PI3K/AKT signalling [38, 39], which results in the suppression of apoptosis and increase in the viability, invasion, and migration of colorectal cancer cells. It was suggested that FN1 could be a prognostic factor and a potential therapeutic target in colorectal cancer [40] and could also serve as a biomarker significantly associated with OS in certain cancers, including BC [41, 42]. In our study, FN1 was identified as a hub gene interacting with ITGB3 and ITGA5 in the PPI network, which is consistent with the study of Bi et all [43]., who found that *FN1* was a common hub gene in different stages (T1–T4) and grades (G1–G3) of BC. KEGG analysis indicated that FN1 was enriched in the PI3K/AKT and focal adhesion pathways, which is in agreement with previous findings that FN1 regulated colorectal cancer spread through PI3K signalling. According to our PPI network, FN1 is predicted to play a role in BC through its interaction with ITGB3 and ITGA5.

Among the top 10 hub genes, LCK was found to be associated with Th1, Th2, and Th17 cell differentiation, T cell receptor (TCR) signalling, and the NF-kappa B pathway, and was closely related to CD4 in the PPI network. LCK is a tyrosine kinase essential for initiating TCR signalling, which can also be involved in signalling through other immune cell receptors [44]. However, the role of the LCK-CD4 axis in BC is unclear. Given that high LCK expression was positively correlated with the survival rate and that the abundance of T cells decreased with the increase of the clinical grade, LCK effects on patient outcome may be associated with its binding to T cells.

Furthermore, we found that 11 immunomodulatory genes known to be involved in cancer immune escape mechanisms were upregulated in tumour samples with high immune/stromal cell scores. Among these genes, 8 showed prognostic potential: 7 (LAG-3, CTLA-4, IFN-γ, ICOS, TIGIT, PDCD1, and KLRC1) were positively and one (ICAM-1) negatively associated with patient survival. Previous studies have shown that CTLA-4 is a critical negative regulator of T cell-mediated immune responses through direct influence on Treg homeostasis [45] and that LAG-3 is linked to metastasis and prognosis of various cancers such as follicular lymphoma, lymphocytic leukaemia, lung cancer, and gastric cancer [46–49].

There are some limitations of this study. First, all patients’ clinicopathological characteristics were obtained from TCGA database and a certain bias due to potential influence of confounding factors such as acute infection, immune system disorders, and anti-inflammatory drugs could not be excluded. As all samples were derived from a retrospective collection, further prospective studies are required to validate the results. Second, the functions of the 97 prognostic genes in the TME were not confirmed experimentally and will need to be independently validated in vitro and in vivo before their use as prognostic indicators in BC. To exclude bias, we plan to address the functional importance of these genes in clinical experiments, which should determine whether their combinations have a higher predictive value than any of them alone.

## Conclusions

Our evaluation of stromal cells and immune cells in the BC microenvironment with the ESTIMATE method provides a new perspective for further understanding of tumour molecular phenotypes. The results suggest that stromal cell scores, ESTIMATE scores, and distinct subsets of tumour-infiltrating immune cells are associated with BC clinical characteristics and outcomes, thus making it possible to identify patients who could benefit from immunotherapy targeting infiltrated immune cells. These results should contribute to understanding of the role of the TME in the progression of BC.

## Supplementary information

**Additional file 1 **Prognostic values of tumour-infiltrating immune cell subpopulations. Unadjusted HRs (boxes) and 95% CIs (horizontal lines) for cases with CIBERSORT *P*-values < 0.05

## Data Availability

The datasets used and/or analysed during the current study are available from the corresponding author on reasonable request.

## Abbreviations

BC: Bladder cancer
TME: Tumour microenvironment
OS: Overall survival;
NK: Natural killer
CTLA-4: Cytotoxic T-lymphocyte-associated protein 4
LAG-3: Lymphocyte-activation gene 3
HAVCR2: Hepatitis A virus cellular receptor 2
PDL-1/CD274: Programmed death-ligand 1
PDCD1/PD-1: Programmed cell death protein 1
IFN-γ: Interferon-γ
ICOS: Inducible T-cell costimulator
ICAM-1: Intercellular adhesion molecule 1
TIGIT: T cell immunoreceptor with Ig and ITIM domains
VSIR: V-set immunoregulatory receptor
KLRC1: NKG2-C type II integral membrane protein
CXCL12: C-X-C motif ligand 12
TILs: Tumour-infiltrating lymphocytes
