# Supplementary material for: In silico analysis of the immune microenvironment in bladder cancer
Source: BMC Cancer. 2020 Mar 30;20:265. doi: 10.1186/s12885-020-06740-5 (PMC7106767; doi:10.1186/s12885-020-06740-5)

Cell	HR	95% CI	P Value
B cells naive	0.958	[0.591–1.555]	0.863
B cells memory	1.027	[0.558–1.888]	0.933
Plasma cells	1.132	[0.697–1.838]	0.618
T cells CD8	1.634	[0.999–2.672]	0.050
T cells CD4 memory resting	0.562	[0.343–0.922]	0.023
T cells CD4 memory activated	1.704	[1.039–2.795]	0.035
T cells follicular helper	1.192	[0.735–1.934]	0.447
T cells regulatory (Tregs)	1.321	[0.814–2.144]	0.260
T cells gamma delta	1.047	[0.589–1.864]	0.875
NK cells resting	1.749	[1.047–2.921]	0.033
NK cells activated	0.915	[0.562–1.491]	0.722
Monocytes	1.031	[0.633–1.678]	0.903
Macrophages M0	0.776	[0.478–1.260]	0.305
Macrophages M1	1.091	[0.673–1.771]	0.723
Macrophages M2	0.904	[0.557–1.466]	0.682
Dendritic cells resting	1.038	[0.640–1.684]	0.879
Dendritic cells activated	1.152	[0.709–1.872]	0.567
Mast cells resting	0.784	[0.481–1.276]	0.327
Mast cells activated	0.972	[0.518–1.824]	0.930
Eosinophils	0.759	[0.414–1.394]	0.374
Neutrophils	0.959	[0.589–1.560]	0.866

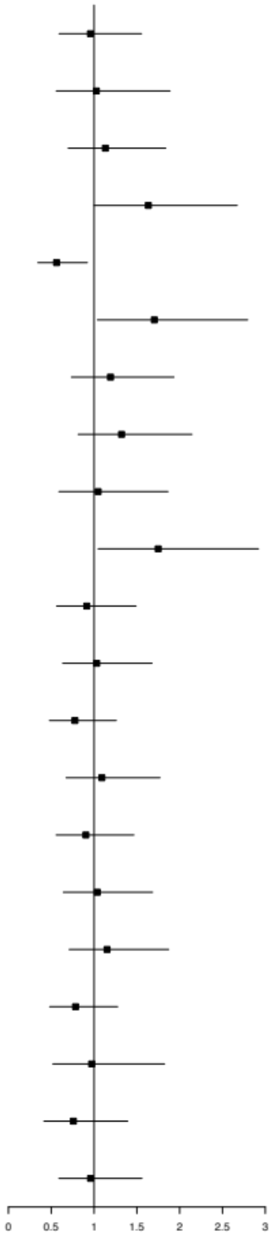

Supplement: Supplementary file 1 [file 12885_2020_6740_MOESM1_ESM.docx]
